# Genome-sequenced bacterial collection from sorghum epicuticular wax

**DOI:** 10.1128/MRA.00484-23

**Published:** 2023-11-01

**Authors:** Marco E. Mechan-Llontop, John Mullet, Ashley Shade

**Affiliations:** 1 Great Lakes Bioenergy Research Center, Michigan State University, East Lansing, Michigan, USA; 2 Department of Biochemistry & Biophysics, Texas A&M University, College Station, Texas, USA; 3 Centre National de la Recherche Scientifique, Laboratoire d'Ecologie Microbienne (UMR CNRS 5557, UMR INRAE 1418, VetAgro Sup), Universite Claude Bernard Lyon 1, Villeurbanne, France; University of Arizona, Tucson, Arizona, USA

**Keywords:** plant microbiome, host microbiota, genomes, bioenergy, agroecosystems, phyllosphere, exudates, dessication

## Abstract

A collection of 44 isolates isolated from the epicuticular wax of stems of energy sorghum is available at the Great Lakes Bioenergy Researcher Center, Michigan State University, MI, USA. We enriched bacteria with putative plant-beneficial phenotypes and include information on their phenotypic diversity, taxonomy, and whole-genome sequences.

## ANNOUNCEMENT

The heat- and drought-tolerant and high-biomass crop bioenergy sorghum (*Sorghum bicolor* L. Moench) accumulates high levels of epicuticular wax on the outer surface of the cuticle to protect plants against various stresses (1–3). It has been shown that the sorghum epicuticular wax harbors specialized microbiome members with putative plant-beneficial capabilities (4). As a resource for genomic and functional diversity of bacteria associated with phyllosphere exudates, we present a collection of 44 bacterial strains isolated from sorghum epicuticular wax. These strains represent 13 families and include 17 genera with putative plant-beneficial capabilities, including nitrogen fixation, phosphate solubilization, resistance to terpenoids, use of methanol as carbon source, and tolerance to desiccation, based on the culture media in which the strains were originally isolated.

Bacterial strains were isolated from the epicuticular wax of stems of bioenergy sorghum hybrid TX08001 grown at the Texas A&M University Research Farm in College Station, Texas (30^o^55′5.55″ N, 96^o^43′64.6″ W). On 2 September 2020, the fifth and sixth fully elongated stem internodes were destructively collected using razor blades into sterile whirl-pack bags. Within 2 hours of collection, the samples were transported to the laboratory on ice and stored at −80°C. On 8 October 2020, samples were delivered to Michigan State University on dry ice and stored at –80°C until processing. Epicuticular wax was carefully scraped from stems with sterile razor blades and collected into sterile 1.5 mL Eppendorf tubes. Wax from different plants was pooled, and 100 mg was resuspended in 1 mL of sterile water. Resuspended wax was serially diluted from 10^−1^ to 10^−4^. To capture a diversity of bacteria from the wax, we used a variety of cultivation media (Table 1). To select for anaerobic bacteria, plates were placed in anaerobic jars containing three bags of anaerobic gas generator (Mitsubishi AnaeroPack System). All plates were incubated at 25°C and 37°C for up to 14 days. Isolated colonies were transferred on new plates with the same medium as used for isolation to confirm purity. Bacteria were grown overnight on R2A broth at 28°C with shaking at 200 rpm, and glycerol stock (25% vol/vol) of pure bacteria isolates was stored at −80°C. Prior to performing whole-genome sequencing, we performed full-length 16S rRNA gene sequencing, with universal primers 27F (5′- AGAGTTTGATCCTGGCTCAG-3′) and 1492R (5′-TACGGTTACCTTGTTACGACTT-3′) (5), of each isolate using the Sanger protocol to link them to corresponding 16S rRNA gene amplicon dynamics in a field microbiome study that was executed at the same field site at Texas A&M University Research Farm (4).

**TABLE 1:** - Taxonomy, colony phenotype, and genome characteristics of 44 bacterial isolates from sorghum epicuticular wax, as described in this study^*a*^

Isolate ID	Bacterial species	Initial isolation media	Temperature, oxygen availability	Colony phenotype	Sequencing platform	SRA accession number	GenBank assembly	Genome length (bp)	*N*^o^ contigs	Coverage	GC %	Genome completeness
SORGH_AS_0981	*Aeromicrobium* sp.	50R2A	37°C,aerobic	Small, white	PacBio	SRP437207	JAVIZD000000000	3,670,284	1	186.7×	25%	98.96%
SORGH_AS_0908	*Pseudoxanthomonas winnipegensis*	50R2A	37°C, aerobic	Yellow	PacBio	SRP437170	JAUTBB000000000	4,331,500	1	82.7×	69.46%	99.53%
SORGH_AS_0909	*Chryseobacterium* sp.	50R2A	37°C, aerobic	Yellow	PacBio	SRP437159	JAVIYY000000000	4,186,940	1	137.1×	40.82%	100%
SORGH_AS_0912	*Acinetobacter baylyi*	50R2A	37°C, aerobic	White, EPS production	PacBio	SRP437185	JAVIZH000000000	3,896,949	3	187.4×	40.23%	99.95%
SORGH_AS_0913	*Nocardioides zeae*	50R2A	37°C, aerobic	White, EPS production	PacBio	SRP437161	JAVIYZ000000000	4,248,407	1	90.2×	73.63%	98.19%
SORGH_AS_0919	*Microbacterium paludicola*	50R2A	37°C, aerobic	Pink	PacBio	SRP437238	JAVIZA000000000	3,351,387	1	196.8×	70.22%	99.24%
SORGH_AS_0974	*Agrobacterium larrymoorei*	50R2A	37°C, anaerobic	White	PacBio	SRP437176	JAVIZC000000000	5,263,363	3	120.0×	58.10%	98.30%
SORGH_AS_0950	*Sphingomonas* sp.	Gauze N-1	37°C, aerobic	Orange	PacBio	SRP437227	JAUTAE000000000	4,389,989	3	73.3×	67.10%	99.14%
SORGH_AS_1204	*Microbacterium* sp.	Gauze N-1	25°C, aerobic	Orange	PacBio	SRP437180	JAUTBD000000000	3,706,564	1	132.3×	70.10%	97.77%
SORGH_AS_1206	*Microbacterium arborescens*	Gauze N-1	25°C, aerobic	Orange	PacBio	SRP437216	JAUTBE000000000	3,449,815	1	88.6×	70.11%	99.24%
SORGH_AS_1207	*Microbacterium trichothecenolyticum*	Gauze N-1	25°C, aerobic	Orange	PacBio	SRP437150	JAUTBF000000000	3,735,270	1	241.2×	69.92%	97.24%
SORGH_AS_0870	*Sphingomonas* sp.	Jensen	25°C, aerobic	Orange	PacBio	SRP437151	JAVIYW000000000	4,339,992	2	98.4×	67.06%	99.42%
SORGH_AS_1048	*Chryseobacterium* sp.	Jensen	37°C, aerobic	Small, orange	PacBio	SRP437220	JAUTAK000000000	4,189,072	1	195.6×	40.80%	99.51%
SORGH_AS_0862	*Microbacterium* sp.	Jensen	25°C, aerobic	Yellow, EPS production	PacBio	SRP437146	JAUTAY000000000	3,504,869	1	174.0×	69.45%	98.27%
SORGH_AS_0892	*Sphingobacterium zeae*	KB	25°C, aerobic	Yellow	PacBio	SRP437175	JAUTBA000000000	5,604,819	1	136.4×	40.08%	99.84%
SORGH_AS_0893	*Acinetobacter baylyi*	KB	25°C, aerobic	White	PacBio	SRP437224	JAVIYX000000000	3,884,413	3	187.0×	40.23%	99.72%
SORGH_AS_0962	*Asaia bogorensis*	KB	25°C, aerobic	Small, Pink	PacBio	SRP437145	JAVIZB000000000	3,299,069	2	112.5×	59.82%	99.71%
SORGH_AS_0969	*Microbacterium* sp.	KB	25°C, aerobic	White	PacBio	SRP437218	JAUTAG000000000	3,939,785	1	159.6×	69.65%	98.53%
SORGH_AS_1094	*Pantoea dispersa*	M9 + 0.4% sucrose	25°C, aerobic	Yellow	PacBio	SRP437173	JAVJAC000000000	4,990,160	3	189.4×	57.57%	99.71%
SORGH_AS_1126	*Agrobacterium larrymoorei*	M9 + 0.4% sucrose	37°C, aerobic	White	PacBio	SRP437223	JAUTBL000000000	4,882,137	2	142.0×	58.10%	97.93%
SORGH_AS_0906	*Klebsiella variicola*	M9 nitrogen free, carbon free	37°C, aerobic	White, EPS production	PacBio	SRP437210	JAVJAI000000000	5,683,212	2	135.4×	57.57%	99.85%
SORGH_AS_1173	*Klebsiella* sp.	M9 nitrogen free, carbon free	37°C, anaerobic	White, EPS production	PacBio	SRP437157	JAVJAD000000000	5,683,290	2	194.8×	57.57%	99.78%
SORGH_AS_1175	*Chryseobacterium* sp.	M9 nitrogen free, carbon free	37°C, anaerobic	Orange	PacBio	SRP437237	JAVIZF000000000	4,186,971	1	211.3×	40.82%	99.88%
SORGH_AS_0842	*Klebsiella variicola*	M9 nitrogen free, carbon free	25°C, anaerobic	White, EPS production	PacBio	SRP437149	JAVJAB000000000	5,683,317	2	89.3×	57.57%	99.93%
SORGH_AS_0878	*Klebsiella variicola*	M9 nitrogen free, carbon free	25°C, aerobic	White, EPS production	PacBio	SRP437219	JAVIZZ000000000	5,683,197	2	188.5×	57.57%	99.55%
SORGH_AS_0879	*Sphingomonas* sp.	M9 nitrogen free, carbon free	25°C, aerobic	Yellow	PacBio	SRP437133	JAUTBJ000000000	4,326,161	2	185.1×	66.43%	99.47%
SORGH_AS_0885	*Nocardioides zeae*	M9 nitrogen free, carbon free	25°C, aerobic	Yellow	Illumina	SRP437487	JAVIZJ000000000	4,252,839	27	353.6×	73.44%	99.85%
SORGH_AS_0887	*Acinetobacter baylyi*	M9 nitrogen free, carbon free	25°C, aerobic	White, EPS production	PacBio	SRP437183	JAUTBK000000000	3,884,591	3	186.4×	40.23%	99.86%
SORGH_AS_0888	*Microbacterium* sp.	MMS methanol	25°C, aerobic	Yellow, EPS production	PacBio	SRP437142	JAUTAZ000000000	3,638,259	1	237.8×	70.61%	98.91%
SORGH_AS_1077	*Microbacterium testaceum*	R2A	37°C, aerobic	Yellow	PacBio	SRP437132	JAUTBC000000000	3,939,786	1	181.0×	69.65%	98.23%
SORGH_AS_1083	*Rhizobium pusense*	R2A	37°C, aerobic	White	PacBio	SRP437166	JAVIZE000000000	5,455,244	3	63.6×	59.10%	99.57%
SORGH_AS_0826	*Klebsiella* sp.	R2A	25°C, anaerobic	White, EPS production	PacBio	SRP437221	JAVJAA000000000	5,685,432	2	136.5×	57.58%	100%
SORGH_AS_0956	*Pseudoxanthomonas winnipegensis*	R2A + 0.1% caryophyllene	37°C, aerobic	Yellow	PacBio	SRP437156	JAUTAF000000000	4,331,831	1	208.2×	69.46%	98.27%
SORGH_AS_0961	*Pseudacidovorax intermedius*	R2A + 0.1% caryophyllene	37°C, aerobic	White	PacBio	SRP437178	JAVLUK000000000	4,187,089	1	241.5×	40.82%	98.77%
SORGH_AS_0993	*Brevundimonas* sp.	R2A + 0.1% caryophyllene	37°C, aerobic	White	PacBio	SRP437129	JAUTAH000000000	2,986,043	1	210.6×	67.51%	99.12%
SORGH_AS_0834	*Klebsiella variicola*	R2A + 0.1% linalool	25°C, aerobic	White, EPS production	PacBio	SRP437138	JAVJAG000000000	5,683,096	2	163.7×	57.57%	99.64%
SORGH_AS_1014	*Xanthomonas sacchari*	TSA	37°C, aerobic	Yellow	PacBio	SRP437228	JAUTAI000000000	4,997,627	2	173.7×	69.65%	98.97%
SORGH_AS_1015	*Roseomonas cervicalis*	TSA	37°C, aerobic	Pink	PacBio	SRP437141	JAUTAJ000000000	4,889,768	5	111.2×	72.05%	98.81%
SORGH_AS_1025	*Klebsiella* sp.	TSA	37°C, aerobic	White, EPS production	PacBio	SRP437181	JAVIZY000000000	5,684,408	2	172.8×	57.58%	99.70%
SORGH_AS_1064	*Chryseobacterium camelliae*	TSA	25°C, aerobic	Orange	PacBio	SRP437167	JAUTAL000000000	4,187,050	1	212.0×	40.82%	99.84%
SORGH_AS_1065	*Siphonobacter* sp.	TSA	25°C, aerobic	Orange	PacBio	SRP437172	JAUTAM000000000	6,150,989	10	125.1×	44.65%	99.70%
SORGH_AS_1067	*Nocardioides zeae*	TSA	25°C, aerobic	Yellow	PacBio	SRP43703	JAUTAN000000000	4,380,404	1	164.8×	73.45%	99.22%
SORGH_AS_1070	*Klebsiella variicola*	TSA	25°C, aerobic	White, EPS production	PacBio	SRP437160	JAVJAH000000000	5,682,994	2	95.0×	57.57%	100%
SORGH_AS_0997	*Pseudoxanthomonas* sp.	TSA	25°C, aerobic	Yellow	PacBio	SRP437165	JAVIZI000000000	4,331,362	1	171.5×	69.46%	99.09%

^*a*^ SRA is the sequence read archive of the U.S. National Institutes of Health.

Individual isolates or the full cryopreserved collections are available. Cultures can be regrown using standard microbiological procedures to transfer a sterile inoculation loop of freezer stock onto the original isolation medium and incubation time (Table 1).

**Fig 1:**
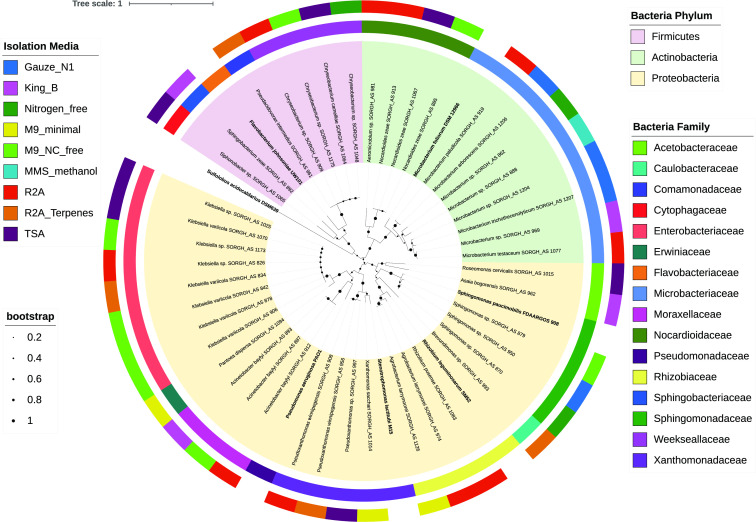
Phylogenetic diversity of the bacterial collection cultivated from sorghum epicuticular wax. The archaea *Sulfolobus acidocaldarius* DSM 639 (ASM2847236v1) was included as an outgroup. The bacterial reference strains *Pseudomonas aeruginosa* PA01 (ASM676v1), *Microbacterium foliorum* DSM 12966 (ASM95641v1), *Stenotrophomonas lactitubi* M15 (ASM280351v1), *Flavobacterium johnsoniae* UW101 (ASM1664v1), *Sphingomonas paucimobilis* FDAARGOS_908 (ASM1602709v1), and *Rhizobium leguminosarum* SM52 (ASM430655v1) were also included. The species tree was inferred from unrooted orthogroup gene trees using STAG and then rooted using the STRIDE algorithms implemented in OrthoFinder. Species tree was annotated with iTOLs.

The high molecular weight genomic DNA of each wax bacterial isolate was extracted by using a phenol/chloroform extraction protocol (6). The genomic DNA of each strain was submitted to the Joint Genome Institute, a U.S. Department of Energy User Facility, for library preparation and sequencing with the Pacific Biosciences (PacBio) platform (7). DNA was sheared to 10 kb using g-TUBE columns (Covaris) and subjected to library preparation using the SMRTbell Express Template Prep Kit 2.0 and sequenced on the PacBio Sequel platform. Samples that failed DNA quality control required by the PacBio pipeline were instead sequenced using the NovaSeq S4 Illumina platform (8). High-fidelity PacBio Circular Consensus Sequencing (CCS) reads >5 kb were assembled with Flye 2.8.3 (9) using default settings. Raw Illumina sequences were quality filtered using BBTools (10). Artifact-filtered and normalized Illumina reads were assembled with SPAdes v3.15.3 (11) (–phred-offset 33 –cov-cutoff auto -t 16 m 64 –careful -k 25,55,95), and contigs were discarded if the length was <1 kb (BBTools reformat.sh: minlength = 1,000 ow = t). Genome completeness and contamination were estimated with CheckM v1.2.2 (12). The assembly was annotated using Prokka 1.14.6 (13), and the phylogenetic tree was inferred from using Orthofinder 2.5.5 (14) and edited with iTOLs v.6.5.8 (15) (Fig. 1).

## Data Availability

Raw sequencing data are available at the Joint Genome Institute’s GOLD database under Study ID Gs0157305, as well as with the National Institutes of Health Sequence Read Archive (see Table 1). Genome sequencing assembly data have been deposited in the GenBank with accession numbers listed in Table 1. The bacterial collection is available at The Great Lakes Bioenergy Research Center at Michigan State University, USA (glbrc@msu.edu).

## References

[1] Xue D, Zhang X, Lu X, Chen G, Chen Z-H. 2017. Molecular and evolutionary mechanisms of cuticular wax for plant drought tolerance. Front Plant Sci 8:621. doi:10.3389/fpls.2017.00621 28503179 PMC5408081

[2] Steinmüller D, Tevini M. 1985. Action of ultraviolet radiation (UV-B) upon cuticular waxes in some crop plants. Planta 164:557–564. doi:10.1007/BF00395975 24248232

[3] Wang X, Kong L, Zhi P, Chang C. 2020. Update on cuticular wax biosynthesis and its roles in plant disease resistance. Int J Mol Sci 21:5514. doi:10.3390/ijms21155514 32752176 PMC7432125

[4] Mechan-Llontop ME, Mullet J, Shade A. 2023. Phyllosphere exudates select for distinct microbiome members in sorghum epicuticular wax and aerial root mucilage. Phytobiomes Journal 7:184–197. doi:10.1094/PBIOMES-08-22-0046-FI

[5] Miller CS, Handley KM, Wrighton KC, Frischkorn KR, Thomas BC, Banfield JF. 2013. Short-read assembly of full-length 16S amplicons reveals bacterial diversity in subsurface sediments. PLoS One 8:e56018. doi:10.1371/journal.pone.0056018 23405248 PMC3566076

[6] Kutchma AJ, Roberts MA, Knaebel DB, Crawford DL. 1998. Small-scale isolation of genomic DNA from Streptomyces mycelia or spores. Biotechniques 24:452–456. doi:10.2144/98243st05 9526657

[7] Eid J, Fehr A, Gray J, Luong K, Lyle J, Otto G, Peluso P, Rank D, Baybayan P, Bettman B, Bibillo A, Bjornson K, Chaudhuri B, Christians F, Cicero R, Clark S, Dalal R, deWinter A, Dixon J, Foquet M, Gaertner A, Hardenbol P, Heiner C, Hester K, Holden D, Kearns G, Kong X, Kuse R, Lacroix Y, Lin S, Lundquist P, Ma C, Marks P, Maxham M, Murphy D, Park I, Pham T, Phillips M, Roy J, Sebra R, Shen G, Sorenson J, Tomaney A, Travers K, Trulson M, Vieceli J, Wegener J, Wu D, Yang A, Zaccarin D, Zhao P, Zhong F, Korlach J, Turner S. 2009. Real-time DNA sequencing from single polymerase molecules. Science 323:133–138. doi:10.1126/science.1162986 19023044

[8] Bennett S. 2004. Solexa Ltd. Pharmacogenomics 5:433–438. doi:10.1517/14622416.5.4.433 15165179

[9] Kolmogorov M, Yuan J, Lin Y, Pevzner PA. 2019. Assembly of long, error-prone reads using repeat graphs. Nat Biotechnol 37:540–546. doi:10.1038/s41587-019-0072-8 30936562

[10] Bushnell B. 2014. Bbtools software package (version 39.01). https://sourceforge.net/projects/bbmap/.

[11] Bankevich A, Nurk S, Antipov D, Gurevich AA, Dvorkin M, Kulikov AS, Lesin VM, Nikolenko SI, Pham S, Prjibelski AD, Pyshkin AV, Sirotkin AV, Vyahhi N, Tesler G, Alekseyev MA, Pevzner PA. 2012. SPAdes: a new genome assembly algorithm and its applications to single-cell sequencing. J Comput Biol 19:455–477. doi:10.1089/cmb.2012.0021 22506599 PMC3342519

[12] Parks DH, Imelfort M, Skennerton CT, Hugenholtz P, Tyson GW. 2015. CheckM: assessing the quality of microbial genomes recovered from isolates, single cells, and metagenomes. Genome Res 25:1043–1055. doi:10.1101/gr.186072.114 25977477 PMC4484387

[13] Seemann T. 2014. Prokka: rapid prokaryotic genome annotation. Bioinformatics 30:2068–2069. doi:10.1093/bioinformatics/btu153 24642063

[14] Emms DM, Kelly S. 2019. Orthofinder: phylogenetic orthology inference for comparative genomics. Genome Biol 20:238. doi:10.1186/s13059-019-1832-y 31727128 PMC6857279

[15] Letunic I, Bork P. 2021. Interactive tree of life (iTOL) V5: an online tool for phylogenetic tree display and annotation. Nucleic Acids Res 49:W293–W296. doi:10.1093/nar/gkab301 33885785 PMC8265157

