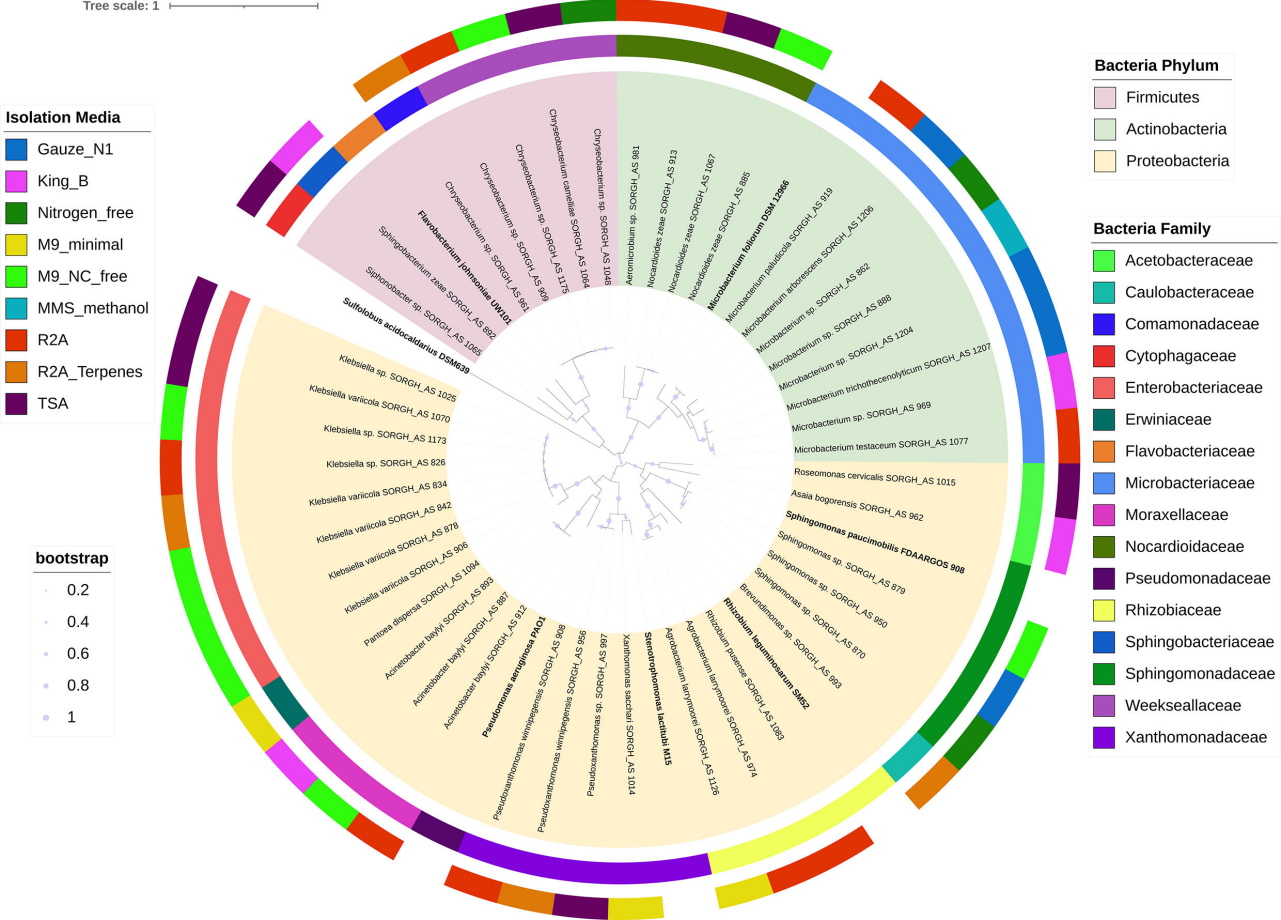# Correction for Mechan-Llontop et al., “Genome-sequenced bacterial collection from sorghum epicuticular wax”

**DOI:** 10.1128/mra.00508-24

**Published:** 2024-07-01

**Authors:** Marco E. Mechan-Llontop, John Mullet, Ashley Shade

## AUTHOR CORRECTION

Vol. 12, no. 12, e00484-23, 2023, https://journals.asm.org/doi/10.1128/mra.00484-23. Page 3, Table 1: Isolate ID SORGH_AS_961 identified as “*Pseudacidovorax intermedius”* should read “*Chryseobacterium* sp.”

Page 4: Figure 1 should appear as shown in this correction.

**Fig 1:**